# A Tale of Two Programs for Parents of Young Children: Independently-Conducted Case Studies of Workforce Contributions to Scale in Bhutan and Rwanda

**DOI:** 10.3390/children10081413

**Published:** 2023-08-19

**Authors:** Frances Aboud, Karma Choden, Michael Tusiimi, Rafael Contreras Gomez, Rachel Hatch, Sara Dang, Theresa Betancourt, Karma Dyenka, Grace Umulisa, Carina Omoeva

**Affiliations:** 1 Department of Psychology, McGill University, Montreal, QC H3A 1G1, Canada; 2 Independent Researcher, Thimphu 11001, Bhutan; karmacdn@gmail.com; 3 College of Education, University of Rwanda, Rwaamagana P.O. Box 55, Rwanda; krwibasira@yahoo.com; 4 FHI360, 2101 L Street NW, Washington, DC 20037, USA; rcontrerasgomez@fhi360.org (R.C.G.); rhatch@fhi360.org (R.H.); comoeva@fhi360.org (C.O.); 5 Save the Children, 899 North Capitol Street NE, Suite 900, Washington, DC 20002, USA; sdang@savechildren.org (S.D.); karma.dyenka@savethechildren.org (K.D.); 6 School of Social Work, Boston College, 140 Commonwealth Ave, Newton, MA 02467, USA; theresa.betancourt@bc.edu; 7 FXB Rwanda, Ruyenzi, Kamonyi District, Kigali P.O. Box 188, Rwanda; gumulisa@fxbrwanda.org

**Keywords:** parenting, implementation research, low- and middle-income countries, workforce, Bhutan, Rwanda

## Abstract

Two case studies of parenting programs, aiming to improve parenting practices and child development outcomes, and implemented by Save the Children/Ministry of Health/Khesar Gyalpo University in Bhutan and Boston College/University of Rwanda/FXB in Rwanda, respectively called Prescription to Play and Sugira Muryango, were conducted by an independent research and learning group. Implementation research focused on the workforce, a crucial but little-studied element determining the success of programs going to scale. Mixed methods were used to examine their training, workload, challenges, and quality of delivery. Health assistants in Bhutan and volunteers in Rwanda were trained for 10–11 days using demonstrations, role plays, and manuals outlining activities to deliver to groups of parents (Bhutan) or during home visits (Rwanda). Workers’ own assessments of their delivery quality, their confidence, and their motivations revealed that duty, confidence, and community respect were strong motivators. According to independent observations, the quality of their delivery was generally good, with an overall mean rating on 10 items of 2.36 (Bhutan) and 2.44 (Rwanda) out of 3. The facilitators of scaling for Bhutan included institutionalizing training and a knowledgeable workforce; the barrier was an overworked workforce. The facilitators of scaling for Rwanda included strong follow-up supervision; the barriers included high attrition among a volunteer workforce.

## 1. Introduction

Recent estimates suggest that over 43% of children under 5 years of age worldwide are not developing their mental capacities as expected for their age [1]. Energized by this statistic, many international organizations have developed and implemented parenting programs to support parents of children in the youngest age group, namely from birth to 36 months. There is now strong evidence that, on average, parenting programs in low- and middle-income countries have a moderate effect on raising cognitive and language development in children when parents attending these programs learn to provide responsive stimulation [2]. In fact, the greatest effects on children are found when the learning for parents focuses on responsive care, specifically the provision of stimulating play and communication in a responsive manner. To promote and support Ministries of Health and other committed organizations in their efforts, the World Health Organization recommended governments support parents in giving responsive care and early learning activities to children in the first three years of life [3]. However, not all programs are effective in passing on benefits to parents and children. The missing piece is often a lack of understanding by service providers and parents of the need for responsive stimulation and how to practice it with children [4]. Because Ministries of Health are responsible for providing services to young children, they have accepted their role in promoting responsive stimulation practices among parents. This entails training and deploying service providers at the community level to reach as many parents as possible [5]. In the meantime, local and international organizations working in LMIC have been training government workers to deliver parenting services to disadvantaged families, and transferring their implementation learnings to the government.

A great deal has been written about the need for implementation data on parenting programs [6]. Likewise, guidelines exist on what is needed to implement at scale and to sustain parenting programs [7,8,9,10]. Yet little actual data are currently available. Most guidelines acknowledge the centrality of the workforce. In this paper, we describe two case studies of organizations implementing parenting programs in Bhutan and Rwanda, and their efforts to scale and sustain a government workforce to deliver sessions to parents with children under 36 months of age.

An effective parenting program has many critical inputs, but, like good theater, it must start with a riveting idea and subsequently be delivered in an engaging manner. A riveting idea delivered badly will not hold an audience to the end. A story with no plot, even if delivered well, will fall flat. So, an effective parenting program must have a curriculum with a compelling account of what parents must provide their children and why, and it must be delivered by a workforce that engages all the sensory, behavioral, social, and emotional brains of its audience. We start with a review of what is known and not known about preparing a workforce.

### 1.1. Workforce: Who They Are

Depending on the context, a parenting program’s workforce can involve health professionals, community health workers (CHWs), para-professionals, or volunteers. The workers delivering the service to parents are critical to its success [9,11,12]. However, training and supervision must be aligned with their basic education level and background knowledge of parenting and child development. Seminal reports by Frameworks [13], based on in-depth interviews with community health workers, reveal that the workforce is often no more knowledgeable than the parents about brain development and responsive stimulation. This applies to health professionals, para-professionals, and volunteers. Training workers in the content of the program (the curriculum) is usually accompanied by training in socially engaging and empathic delivery skills. Although this is regarded as the most important support given to parenting service providers, it is by no means the only one: a workforce with little initial knowledge on this topic will continue to need support in the form of refresher training, monitoring for quality, and mentoring through supervision. The amount of required training and supervision likely decreases as professionalism increases, yet health professionals are already overworked. Consequently, para-professionals (e.g., community health workers) and volunteers are being given this task as part of a general trend toward task-shifting and -sharing. The professionals include university-trained paid health workers tasked with delivering medical services and health education. Village volunteers may have some training for specific health campaigns and child protection, and are familiar to families, yet they often receive little compensation except social recognition. Little information is currently published about status implications for training, workload, supervision, quality of delivery, and motivation for the work. Our two cases highlight similarities and differences between health assistants, a professional workforce providing parenting programs in Bhutan, and village volunteers used in Rwanda. There are benefits and challenges to both types of workers when it comes to delivering a program that is both scalable and sustainable.

### 1.2. Workforce Preparation for Parenting

Tomlinson [14] and others [15] have discussed the selection, training, and supervision of the frontline workforce. It is likely that workers who have good counseling skills and empathic personality dispositions will be more effective in changing parental practices. However, many programs do not interview and recruit suitable job candidates. Rather, the community may have its own strategy, for example, to select highly respected mothers or CHWs living in the community, or they may already have an assigned health professional. Regardless, preparing the workforce to deliver a parenting program requires many days, often beyond what a Ministry of Health allows. Tomlinson [14] also suggests that refresher training is needed for the continuous development of delivery skills by non-professionals. Finally, supervision must go beyond administrative accountability to include observation of the delivery, feedback on quality, and reflective mentoring. Hill and others [16] looked at the impact of supervision variables on implementation outcomes such as coverage, performance, and perception of quality. The supervision variables were frequency, mode (peer, group, and community), tools (self-assessment and checklists), focus on quality assurance or problem-solving, and training. Unsurprisingly, the quality of supervision (supportive, rather than record-taking) enhanced CHW performance more than its frequency. The two case studies described here differed in their supervisory support to workers and this will be measured using independent observations and interviews to glean the perspectives of workers.

Currently, no common method exists to monitor the quality of delivery of a parenting program [17]. However, there is some consensus on the necessary skills, most of which are both critical and observable. Tomlinson [14] outlines five main skills needed by a worker to deliver a parenting program: (1) communicating the main issue, in this case, that responsive stimulation importantly starts at birth; (2) communicating the necessary information, for example, that children’s brains are ready to hear language and interact playfully at birth, and that children need time every day to interact with family members; (3) helping parents build parenting skills through practice and active learning, e.g., showing how to talk responsively to an infant; (4) solving problems that parents encounter when intending to enact the practices; and (5) helping parents strengthen their social supports. These elements can be detailed in a manual for workers to use when delivering a session. To these must be added a set of social delivery skills: showing acceptance and empathy when parents struggle with new practices, facilitating an honest discussion of problems while eliciting possible solutions, and being gentle and respectful when providing feedback. “Caring” is the term used by Shankar to refer to the social skills of listening, empathy, and praise; the concept is otherwise known as “heart” in the tripartite “head, heart, and hands” [18]. The manual can outline the “head”, i.e., knowledge about child development and parenting, and “hands”, i.e., practical activities concerned with changing parenting practices through an active learning experience. Face-to-face training is needed for “heart” skills [19]. Some evidence confirms that these delivery qualities are associated with greater changes in parenting practices [20].

Based on publications outlining features of the workforce that are critical to maintaining quality at scale [9,14,15], we developed four measures. The training of the workforce was observed and rated using a multi-item list of content, delivery skills, and methods used to train them. A workforce survey inquired about quantifiable details of their training, supervision, and workload. In-depth interviews examined workers’ knowledge of child development, parenting, ways to change parenting practices, and challenges. Observations of important qualities of delivery revealed the skills workers used to interact with parents. The goal was to examine workers’ engagement from training to delivery, sourcing both the perspectives of workers and of an independent observer.

### 1.3. Programmatic Features

The nature of a parenting program is as important as the workforce delivering it [7,12]. For example, the intensity or dosage of contact with parents may be critical: it may need to be longer for parents who are not yet engaging in responsive stimulation, though this is not confirmed with evidence. Groups may be more cost-effective than home visits, but this too requires more evidence [21]. The inclusion of behavior change strategies built around active learning may be necessary. In contrast to a didactic approach, where parents are counseled without their children present, recent effective programs use demonstration and coaching as parents practice new ways to play and talk with their children. When the curriculum is embedded in a structured manual of activities organized by session, available for training and delivery by inexperienced workers, consistency and fidelity can be maintained. Our two cases differ on some of these programmatic features, making them somewhat incomparable yet instructive when presented together.

### 1.4. The Context for This Research

The two parenting programs presented here are Prescription to Play (P2P) implemented by Save the Children/Ministry of Health/Khesar Gyalpo University of Medical Sciences in Bhutan (and based on their Building Brains Common Approach), and Sugira Muryango (SM) implemented by Boston College/University of Rwanda and its partner organization FXB Rwanda. Both are funded by the LEGO Foundation and began scaling their program in 2020 following successful evaluations [22,23].

Some information about the design of their programs helps to lay the groundwork for an understanding of their workforce. Each had created a parenting program for its respective country, built on existing programs, and had reasonable data showing that their program was effective. Both intended to scale it out over 3–4 years to include many more families—56,000 families in all 20 districts of Bhutan and 10,000 in 3 districts of Rwanda. Both had plans to engage the government by using an existing workforce: health assistants in Bhutan who are part of the Ministry of Health system, and Rwandan Friends of the Family (inshuti z’umuryango or IZUs) who are recruited by the Ministry of Gender and Family Promotion and regulated by the National Child Development Agency. Finally, both provided their workforce with a manual detailing activities for 12 sessions. In other respects, their initial designs were different [7]. Bhutanese parents (here the term “parent” is used to include any “caregiver”) were recruited from mainly rural families who are registered with the nearest Primary Health Center for their child’s basic health care; on the other hand, Rwandan families were a targeted group participating in the government cash-for-work program implemented for households ranked by the government as living at the most extreme level of poverty. The Bhutanese workforce were professionals on a government salary, whereas the Rwandan workforce were volunteers who received incentives for attending training and limited in-kind incentives from the government (transportation and cell phone top-up). A group delivery modality was used in Bhutan, whereas home visits were delivered in Rwanda.

Funding from the LEGO Foundation was related to the inclusion of play and nurturing care in their curriculum. The Bhutanese program included 12 monthly sessions delivered to groups of parents and their children; nine of these sessions emphasized play and communication, such that four games for different age groups were demonstrated and practiced at each, using household materials like cups, cloth, and pictures. The Rwandan program also included 12 home visits, but they were delivered weekly along with 3- and 6-month booster sessions that reviewed earlier topics. A total of 4 of the 12 included demonstrations of new ways to play and talk to children, though 15 min during each visit was devoted to parents showing how they played with their child while being coached by the worker. The themes of the non-play sessions in both programs included nutrition, hygiene, stress management, family conflict, and discipline, with some attention given to play at each session.

An independent research partner, FHI360, was engaged and funded by the LEGO Foundation to collect data on the implementation process of each program as they began to scale up. This was done through a collaborative arrangement with the implementing partners (Save the Children and Boston College) so there would be no duplication of data collection. Scaling up is defined as “deliberate efforts to increase the impact of [parenting] innovations successfully tested in pilot or experimental projects so as to reach and benefit more individuals and to foster policy and programme development on a lasting basis” [24]. The ExpandNet framework for scaling health programs identifies what can be measured and tracked over time [24]. Interviews with the resource team, namely the implementing partner, and desk reviews of their innovation, specifically the parenting program, set the stage for an examination of how an extensive workforce would be trained and supervised to maintain quality at scale. Concerns of government stakeholders regarding human resources were also explored in annual in-depth interviews but not reported here. The data were expected to fill a gap in our understanding of how parenting programs transition to scale and the challenges they face in maintaining the quality of delivery.

The overall objective was to describe the training, supervision, and functioning of the workforce through surveys, in-depth interviews, and observations. Based on data from Year 2 (2021–2022) the research objectives were as follows:

1. Describe the workforce training in terms of parenting topics covered, delivery skills addressed, and methods of teaching;
2. Describe in quantitative form the training, supervision, workload, and parenting knowledge of the workforce in each country;
3. Describe in qualitative form the workers’ understanding of the challenges they faced while facilitating parenting practices to promote child development, and how well prepared they felt after training and supervision;
4. Evaluate the quality of delivery by the workforce in terms of the use of active learning methods and parent participation.

## 2. Materials and Methods

Suggested methods for assessing workforce competence and delivery quality were considered with the understanding that most are not standardized [25]. The CARE reporting framework for the implementation of early childhood development programs was used to identify constructs and how to report the measures and findings [26]. Ethical approval was obtained from ethics committees at FHI360, McGill University, and Ethics Review Boards in Bhutan and Rwanda. Particular attention was paid to maintaining the anonymity and confidentiality of the data so workers would not feel that their job was in jeopardy by participating in the research.

### 2.1. Participants

The participants were the frontline workers who were trained and who delivered the parenting programs directly to parents with their children, namely health assistants in Bhutan and Inshuti z’Umuryango (IZU)/Friends of the Family volunteers in Rwanda. Informed consent was obtained from each worker (also referred to as a provider) at the time they were interviewed or observed. Data were collected while providers were trained and then later in the midst of delivering the programs to parents, which started in early 2022 in Bhutan and 2021 in Rwanda—both delayed somewhat due to COVID-19 restrictions.

The training was observed on multiple days during the 10–11 days of initial training of a cohort of the workforce. Training was conducted by Save the Children for the Prescription to Play program in Bhutan, and by FXB Rwanda for the Sugira Muryango program. The particular cohorts observed were selected based on convenience ahead of the program being implemented, and it was reasonably assumed that the same agenda and methods of teaching/learning would be used for other cohorts.

Workers for the phone survey were chosen at random from the pool of recently trained providers. The sample constitutes a majority of recently trained workers in each country and was based on an expected improvement of 5 points out of 40 (1SD) in the knowledge test over time, leading to a required sample of 128.

A purposive subsample of ten to twelve recently trained workers, who varied in gender and educational background, was selected to participate in in-depth interviews. Saturation was reached with this number and the intention was to follow up the same workers over time. Finally, observations were made of 60 different randomly selected providers in each country, using the phone survey as a sampling frame, as they conducted a group or home session. In Bhutan, providers were approximately 8 months into delivering their monthly program, whereas in Rwanda they were delivering the 3-month booster visit.

### 2.2. Measures and Procedure

Table 1 outlines the four methods used to gather information on components of workforce training, supervision, and support, along with providers’ perspectives on these components. Sample sizes and dates of data collection are included. Details on each measure follow. Copies of measures may be requested from the authors.

#### 2.2.1. Observation of Training

A structured observation measure was used to identify the parenting topics covered, social skills practiced, and teaching/learning methods used by the trainers to help workers learn how to deliver a session to parents. To create the items to be observed, we consulted experts involved in training professionals, para-professionals, and volunteers for previously known and effective parenting programs. Because we were interested mainly in parenting for child development, topics related to play, communication, milestones of development, and father engagement were recorded, but not nutrition and health, though they also may have been taught. Skills for delivery included, among others, showing respect and empathy, listening and responding to parents, giving gentle feedback, and engaging parents. Methods of teaching included observation of a delivery enacted by a trainer or on video, role-playing delivery with other trainees, receiving feedback on delivery from the trainer or trainees, didactic teaching, and group reflection on one’s performance. These methods were rated during multiple 2–3 h blocks of time in order to determine their approximate frequency. Observers recorded their observations on the Kobo Collect app, and these were forwarded directly for analysis; if an item is recorded as observed, a space opens on the app for extra descriptive comments to be written. Observers were trained by an international and a local researcher over three days during which detailed descriptions were given for each item. Inter-rater reliability against the local researcher reached over 80% for the first training attended.

#### 2.2.2. Phone Survey of Workforce

The structured survey consists of 20 questions with sub-questions. It includes demographic variables (age, sex, years of education, and years of experience); workload for this program and for other duties; training and supervision; confidence in delivery; perspective on own work; and the 20-item Knowledge of Child Development Inventory assessing knowledge of child development with 10 items and ages for appropriate provision of stimulation with 10 items [27]. The survey items were derived from papers on workforces [16] with relevance to our two parenting programs. Enumerators at a local data collection firm were hired and trained by an international and a local researcher to deliver the survey by phone. Up to three attempts were made to reach each interviewee; if this was unsuccessful, an alternative person on the list was recruited. Answers were recorded on the Kobo Collect application by the interviewer and analyzed using descriptive statistics. A second interviewer repeated 10% of the surveys a week later to assess consistency.

#### 2.2.3. In-Depth Interviews

The semi-structured interview guide requests detailed information on providers’ understanding of what their work entails. Questions are open-ended and focus on issues that arose from the phone surveys. Items include parenting practices they attempt to change and how; workers’ understanding of the terms “early child development” and “responsive stimulation”; challenges they encounter in their work; most effective methods used in their own training; supervision and peer support; their motivation for work; and the perceived demand for their program in the community. Local researchers who are part of the research team conducted the interviews by phone. It took approximately 40 to 60 min per interview. The interviews were audio recorded, transcribed, and translated. Both the local researcher and the lead international researcher read through the transcripts and conducted a content analysis; the former wrote the first draft of a summary, including representative quotes.

#### 2.2.4. Delivery Observations

Based on previously published measures of delivery quality [18,20], qualities found to be important in changing parenting practices are listed. Ten qualities concern specific activities related to active teaching/learning, such as introducing a new play or communication activity, demonstrating the activity, giving parents an opportunity to practice the new activity with their child, and coaching. If not observed, the item is scored 1; if observed but delivered in a cursory, confusing, or inadequate manner, it is scored 2; if observed and delivered in a very good manner, it is scored 3. Four additional items concern qualities that can be observed throughout the session: if the provider appears to be prepared, covers the content of the session, delivers it in an engaging manner, and expresses acceptance and empathy toward parents. These are scored 1 (poor), 2 (inadequate, could be improved), and 3 (very good). Three qualities of parent participation [28] are noted as yes or no for home visits and as few (25%), some (50%), or many (75%) for group sessions: parents report engaging in the new practices since the last contact (homework); parents practice activities with their child during the session; and parents have their own play objects available during the session. For training purposes, a more detailed description of each quality is provided to data collectors.

The training of observers from the data firm was conducted over three or four 3 h sessions using written vignettes of hypothetical sessions based on the parenting program being delivered. Trainees were required to rate every instance they saw in the vignette that reflected one of the qualities to be observed. Over the course of three separate assignments, inter-rater reliability with the trainer reached 80% and above for all observers. During the first two days of observations, observers in pairs independently rated and submitted their data for the same session, then discussed the discrepancies to arrive at a consensus, and submitted the consensus ratings. These independent paired ratings also surpassed 80% agreement.

## 3. Results

Results for the four methods of data collection are presented in descriptive form. Data were collected by independent data firms and researchers in each country, and all analyses were conducted by independent analysts. Although the intention is not to statistically compare the two programs, similarities and differences are highlighted.

### 3.1. Observations of Training

Both Bhutan and Rwanda partners were observed to use a manual for training and delivery, with detailed descriptions of the activities to be performed in each session. These activities formed the basis of training. Two to three trainers conducted the training of 15 to 20 trainees lasting approximately 10 days—11 were observed in Bhutan and 10 in Rwanda. Not all days were devoted to parenting for child development: Bhutan had 9 out of 12 sessions on play and communication, developmental milestones, and respectful discipline; Rwanda had 4 out of 12 sessions on play and communication, with others devoted to family conflict and stress management. Both addressed hygiene and nutrition to an extent. Neither used video, but Bhutanese trainers had playthings and books for providers and caregivers to use with children during sessions along with illustrated play cards to take home. The Rwandan program emphasized the use of homemade playthings; trainees were shown how to make them and thereby engage caregivers to make their own playthings out of locally-sourced material.

**Figure 1:**
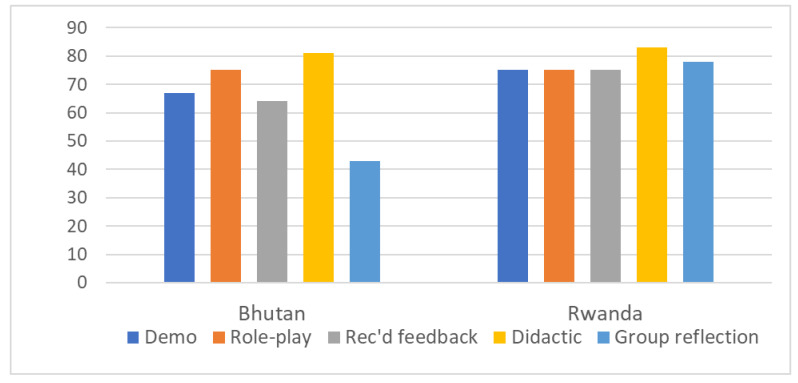
Active methods of teaching and learning: percent of time blocks where the method was observed. Notes: demo means that trainees watched trainers demonstrate provider–parent interactions; role-play means that trainees practiced delivery with each other; received feedback refers to feedback on role-playing from a trainer or from peers; didactic means lectures or reading from the manual together or separately; group reflection refers to trainees evaluating their own learning and performance.

Figure 1 presents the frequency of using various methods of teaching trainees during the parenting-for-child-development sessions, expressed as the percent of 2–3 h time blocks. Close to 80% of time blocks used role-playing by trainees and didactic reading or lecture-type presentations by trainers. Demonstrations by a trainer of how to deliver a session and feedback on trainees’ performance were similarly frequent—slightly more so in Rwandan training. Demonstrations, role plays, and feedback are considered powerful methods by which to learn how to deliver sessions [12,14]. Rwandan training included the frequent use of group reflection in which trainees evaluated their performance and learnings.

The delivery skills listed in Table 2 were addressed in the large majority of observed training sessions. The lowest frequencies were found for cautioning trainees to address parents rather than children as their main audience, and to explicitly probe parents for difficulties they encounter when trying the new practices.

### 3.2. Survey of the Workforce

According to the results presented in Table 3, health assistants in Bhutan were part of the government health system and had higher levels of education and longer experience in their position compared to the volunteer IZUs in Rwanda. The former were also more likely to have extra COVID-19-related responsibilities and to feel overworked (80% compared to 8% in Rwanda); yet both felt respected and appreciated for their parenting work. Health assistants had responsibilities in the clinic, whereas IZUs conducted other child protection activities in their village. To deliver the parenting program, the health assistants in Bhutan met with approximately two groups of 20 families per month (pro-rated to 5 families per week), whereas IZUs in Rwanda visited on average 2.6 families per week. The latter had been conducting the parenting program for 2 months, meaning they had delivered approximately 8 of the 12 modules, whereas the health assistants had delivered approximately 4 monthly modules.

Days of training were similar, but follow-up support was stronger in Rwanda where most had received refresher training, recent face-to-face supervision, and peer support. Confidence in delivery was high, with an average quality rating between 7 and 8 out of 10, according to providers. Yet on a 40-point Knowledge Inventory, the means hovered around the midpoint, higher for health assistants, as expected. The mean for Turkish mothers who had no training in parenting was 19.2 [27]. A common error was to make later-than-expected attributions, leading parents and providers to offer stimulation later than required for mental development.

### 3.3. In-Depth Interviews

Data from the two countries are presented separately in order to convey the personal perspectives of the providers. Quotes from interviewees are included without identifying information because of the small sample.

#### 3.3.1. Bhutanese Health Assistants

Bhutanese health assistants clearly understood the centrality of brain development and how it was related to mental capacity and behavior. They understood the term “stimulation” and the role of parents playing with their children, but most understood that children would be responsive to parents rather than vice versa. When asked: “What exactly do you tell parents that influences them to adopt new play practices? What exactly do you do?” they said by advising, showing, demonstrating, and talking about the benefits of play. Some talked about specific games and songs they teach, and they emphasized to parents the specific skills acquired with each activity.

“….we tell them that playing at stacking cups helps develop motor skills, while singing “kuzuzangpo” song teaches them tunes, words and also to associate the words to greeting someone and bowing slightly when you meet.”

Challenges in persuading parents included a lack of interest in the new play leading to a lack of attendance at group sessions and disinterest in practicing the games during sessions and at home. Fathers and grandparents were also said to express disinterest. Challenges in conducting sessions included finding time in their busy schedule along with a convenient space, and being called to attend health campaigns.

Perspectives on their training revealed strong support for the materials received to help them deliver the program, including the step-by-step outline of activities presented in the structured manual. Sitting mats, toys, books, and visual charts were also helpful during training and delivery. Supervision by a District Health Officer was rare except perhaps on a national- or district-level social media platform where supervisors checked to see that sessions were being conducted and to offer advice. Especially in more remote areas, face-to-face monitoring did not occur. Planned meetings with other providers were infrequent, but those who met up spontaneously in a health center said the exchanges were useful.

“It is very helpful as we share each other’s experience in group sessions and what one does when we encounter problems. That way we are exposed to new alternatives for solutions. This also helps in getting information on preparation.”

When asked what motivates them to do this work, they mentioned the value of the program for parents and children, and its recognition as an important component of the health system. Health assistants were also paid workers.

As part of a question on whether the program would be sustained once external funding finished, we asked about the demand for the program. The respondents felt that demand required effort to sustain at the community level. They suggested that the government might need to initially incentivize families to attend, by providing oil or rice, the way they had done with the immunization program, which now no longer requires incentives.

#### 3.3.2. Rwandan Child Protection Volunteers/Inshuti Z’umuryango (IZUs)

Rwandan IZUs had a clear understanding of the phrase child development as encompassing cognitive, emotional, and social development and the brain. However, they were less clear about the term “responsive stimulation”, describing ways that parents could provide stimulation most of which were instructional rather than responsive. When asked: “What exactly do you tell parents that influences them to adopt new play practices? What exactly do you do?”, they listed a number of specific play activities along with verbal persuasion by citing the benefits for their child.

“Play ensures that our bodies make movements, enhances sociability, creates bonds with parents; helps the brain to be active” and “The child does not become lonely… If the child is social, it is easy for a teacher to teach the child when he starts school.”

Challenges in persuading parents to change their practices included not seeing the importance, being too occupied finding food and other material supplies, conflict between parents, and norms around fathers’ engaging with their children. “Parents do not see the importance of playing with a child. They see it as waste of time”. They solved this by working on trust, and being available to help families solve problems. Challenges in delivering the sessions included the lack of basic materials among poor families such as soap and materials to make playthings and having to delay the visit if the family was out working or otherwise occupied.

Perspectives on training emphasized the value of their manual and its illustrations, which was used to prepare for each visit; preparation time varied between 20 and 60 min per family. They also referred to training in how to be humble with poor families.

“The teaching manual was well prepared, like when teaching on a certain lesson I would check in the manual and understand well what to train about. The learning became concrete. Everyone should have this teaching manual in their homes.”

Supervision took place weekly and often a supervisor watched a home visit and gave feedback. They also appreciated weekly meetings with other providers in order to exchange experiences and solutions to problems, and to arrange to fill in for a sick colleague.

“We all needed to learn and refresh our minds. Because there were things I was doing wrong … We educated each other and corrected each other on how best to teach families.”

Given that the Rwandan providers were volunteers, it was useful to hear what they said about their motivations to do this work. Some said that they were motivated by the changes they saw in families due to their influence: “to see families improve their lives… a sense of worthiness”. Others said the solutions they discussed with families were relevant to their own lives: “These courses are relevant to me and my life as well. I learn as I teach. I have improved and now able to solve my own problems as well”. Others said it was their duty as an IZU and because they had been trained and supported: “I accepted the responsibility because of the support I was given to do the activity”. They all agreed that they would continue with their work if the government decided to take over the program.

A question regarding the demand for the parenting program was answered most often in terms of need. However, demand was reported to increase among families once they saw the benefits of the program, and among others who were in the second SES quintile and therefore did not receive the program. Local leaders were also reported to increasingly express a demand for the program.

### 3.4. Delivery Observations

Delivery by health assistants in Bhutan received a mean of 2.36 out of 3 on the ten qualities related to active teaching/learning (see Table 4). Only a few were not observed at all; most received a score of 2 for “needs improvement” and several such as “introduces new play” and “explains benefits for child” received a score of “very good”. In terms of delivery skills, most covered the intended content of the session but were less engaging than expected. In terms of parental involvement, it appeared that “few” or “some” (more than 25% but less than 75%) had practiced the new activities during the intervening month, actually practiced the new games with their child during the sessions, and had their own playthings.

Delivery by IZU volunteers in Rwanda received a mean rating of 2.44 on the ten qualities (see Table 5). Once again, very few activities were not observed at all; most were performed very well by over half of the volunteers. Delivery skills were observed to be very good in over two-thirds of the home visits and parents practiced with their child using their own playthings. Over 98% of parents did their “homework” by engaging in play with their children between sessions.

## 4. Discussion

Health assistants in Bhutan delivered the 12 monthly sessions to groups of parents with their children, whereas the Rwandan volunteers delivered 12 weekly home visits to parents and children. The first challenge was that both were similarly unfamiliar with brain development and parenting for child development ahead of training for this parenting program. They also noted that parents initially did not see the benefits of responsive play and communication with children. However, they received similar 10–11-day training with a manual to guide them through delivery. Follow-up support was stronger for the Rwandan IZUs in terms of refresher training, supervision, monitoring of their visits, and peer exchanges. Yet, much of this support for delivering parenting visits would disappear once the 12-week program was over. Furthermore, as volunteers, they received little remuneration. Challenges for the Bhutanese health assistants included a lack of follow-up training, monitoring of their group delivery, peer exchanges, and a heavy workload, which is a common complaint among professionals. To accommodate their workload, the health assistants delivered two group sessions a month but long intervals between sessions and the need to send out reminders to parents made parent attendance challenging. Regardless, providers in the two countries received high ratings for the quality of their delivery, especially in terms of using techniques central to active learning for parents. Health assistants were insufficiently lively and engaging—a more difficult but much-needed skill when addressing groups—and this was reflected in less engagement in play activities on the part of parents. We caution against drawing strong causal or comparative conclusions as the study design was intended to be descriptive, and the modality of delivery and target beneficiary families differed in the two settings.

These findings are now discussed in greater detail as they pertain to critical issues in preparing and supporting a workforce to deliver effective parenting programs at scale. Mixed method data from the two case studies reinforce previous guidance on scaling horizontally throughout a system and add new insights. They are based on the recognition that providers being used by most Ministries of Health in low- and middle-income countries, whether volunteer or professional, are unlikely to have experience or expertise around parenting for child development, much like the parents they are training. Professionals are conveniently part of the system, yet are already overburdened in attending to other duties, while volunteers who are trusted members of their communities will show higher attrition unless compensated. Going to scale requires that the workforce be continuously expanded while maintaining a consistently high level of quality, perhaps by institutionalizing the parenting program and training within the health system. As a program expands, different forms of monitoring for quality may be required. Going to scale also requires built-in feedback loops whereby implementation data are used to improve delivery.

### 4.1. Continuous Development of the Workforce

If “scaling” involves expanded reach and benefiting more individuals [24], there needs to be expanded training of a workforce in many regions of a country, and a workforce that will be retained. The professional health assistant cadre in Bhutan appears to be well-suited for retention and scale-up and is currently receiving pre-service training at the university. The Ministry of Health has institutionalized their parenting duties along with clinic duties, paid them, and recorded their activities in the health information system. There is a less clear pathway for the workforce in Rwanda, made up of unpaid volunteers under the Ministry of Gender and Family Promotion; they are regulated by the National Child Development Agency which oversees many such projects aimed at child protection and development. Scaling across the country in this case might involve creating coalitions of different early childhood programs operating in different districts.

### 4.2. Maintaining Consistent Quality

Both programs showed high quality both in terms of the ten critical activities needed to encourage behavior change among parents and in their delivery of social skills. The health assistants received little follow-up support after their initial training, while the Rwandan volunteers received strong supervision from mentors and peers. Maintaining quality as the program scales up across the country requires such supervision.

In other respects, the programs in Bhutan and Rwanda both had two features that would facilitate consistent quality: one is the use of a detailed, structured curriculum manual highly appreciated by providers both during training and when preparing to deliver sessions to parents. Manuals are also essential for consistent scaling up across regions in a country [9]. The second feature that facilitates consistent quality is the use of active teaching/learning methods when training workers and when workers train parents.

To maintain quality, competence after training should be assessed. Of the multiple methods suggested for assessing competence [25], only an assessment of knowledge about child development and parenting was included here [27]. More important would be an assessment of delivery performance. The latter could be assessed by having trainees deliver a mock session and be rated using a monitoring tool. Trainees who do not meet standards would be apprenticed for a period to another worker who performed well.

Only the Rwanda program had an ongoing system for monitoring the quality of providers’ home visits. It required the volunteers’ supervisor to observe, rate, and provide feedback. The Bhutanese program has now put one in place using a local organization. Many parenting programs use such a system, including the Cuna Mas program in Peru [29]. Others have used videos of the home visit that are rated later for quality. Alternatively, members of a village committee could observe and evaluate the quality of a visit. Regardless, feedback with some form of mentoring is essential. Few measures of quality exist outside of high-income countries, but those that do include many of the qualities we used here [29]. Although some adaptation needs to be considered, especially if the curriculum is not as structured as the ones observed here, many of these qualities are central, and have been validated against changes in parental practices [20].

### 4.3. Feedback Loops: Using Implementation Data about the Workforce to Improve Delivery

If scaling up requires “deliberate efforts” to expand the reach and improve the outcomes of a parenting program, then these efforts must be based on implementation evidence [9]. In many cases, the feedback comes too late, as was the case for the Pelotas 15-year scale-up; high turnover among the workforce and a reduction in delivery quality resulted in no significant benefits for the children [30]. The findings presented in our study were fed back to implementing partners in a timely manner, but it was their decision to use the evidence as they wished. Both did so. In Bhutan, the implementers re-trained health assistants to improve low-scoring qualities such as problem-solving with parents and being responsive by following the child’s lead; they embedded delivery qualities in their monitoring form and in-service training. Likewise, the Rwandan implementers refreshed volunteer providers and developed a dashboard of quality items for independent supervisors to use when observing home visits. Delivery quality is the most impactful output in the sequence of implementation measures and findings to be reported [26] in that it can be associated with parenting practices [20]. Delivery quality data are likely to trigger changes in workforce training and possibly the design of the curriculum, for example, making it more structured if providers and parents need more guidance.

### 4.4. Limitations

One limitation is the lack of any statistical comparison of the two cases. Rather, we simply highlighted similarities and differences that were of interest. Statistical comparisons along with validation against endline changes in parental practices would be important. The methods for collecting quality data differed in that they were collected by an independent data firm in Bhutan and by potentially biased supervisors in Rwanda. Although supervisors’ reliability with a local research firm was high, supervisors might have deviated once the reliability test was finished. Potential biases in self-reports from the workforce when answering questions from the survey and in-depth interviews is a well-known limitation, but one that is acknowledged and accepted when one wants to understand the perspective of providers. The generalizability of the findings is limited by the nature of the workforce, their delivery modality, the families they intended to benefit, and more generally, the countries in which they lived and worked.

## 5. Conclusions

This paper presented two case studies of parenting programs developed by organizations in Bhutan and Rwanda and studied by an independent research and learning group. The implementation research focused on the workforce, arguably the linchpin of a successful program. Expanding the workforce as the program scales horizontally and vertically requires a mechanism to institutionalize training and to build in continuous quality improvement and workforce skills development. Data on the quality of delivery, because they are known to be associated with parenting outcomes, are most likely to trigger changes in training and in continuous development through supervision. In recognition of the value of quality data, both programs described here instituted regular monitoring of program delivery using a robust quality tool, similar to the one applied here, early in the case of Rwanda and later in the case of Bhutan. In addition to independent assessments of training and delivery, new insights were gained from the perspectives of the workforce. Their own assessments of the quality of their delivery, their confidence, and their motivations revealed that duty, confidence, and community respect were strong motivators. These motivations can drive an existing workforce with minimal experience in early child development to manage workloads, share tasks, and deliver early childhood programs with quality. Continued support for the workforce in terms of refresher training, mentoring, and peer coaching is advisable to maintain quality implementation at scale. From the workers’ perspective, sustaining a quality program additionally requires financial and policy support from the government.

## Figures and Tables

**Table 1:** Measures, sample sizes, and dates when administered.

Measures	Bhutan Prescription to Play	Rwanda Sugira Muryango
1. Observation of Training		
Date of data collection	September 2021	April 2021
Sample size	*n* = 16 days	*n* = 15 days
2. Survey of workforce		
Date of data collection	June 2022	July 2021
Sample size	*n* = 106	*n* = 150
3. In-depth interviews		
Date of data collection	July 2022	December 2021
Sample size	*n* = 10	*n* = 12
4. Observation of delivery		
Date of data collection	September 2022	January 2022
Sample size	*n* = 60	*n* = 60

**Table 2:** Delivery skills addressed during training.

Delivery Skill	% of Training Sessions Where Observed	
	Bhutan Prescription to Play	Rwanda Sugira Muryango
Clarify objectives for parents	100.0	100.0
Engage parents	100.0	100.0
Give opportunity for parent–child interaction	93.7	100.0
Show respect, empathy, and confidentiality	100.0	73.3
Listen and respond to parents	100.0	66.7
Probe parents regarding difficulties	81.2	53.3
Praise	93.7	100.0
Provide gentle corrective feedback	87.5	93.3
Interact with parents (less with child)	62.5	66.7

**Table 3:** Survey results.

Variable	Bhutan Prescription to Play	Rwanda Sugira Muryango
Demographics		
Current status	Health Assistant	Child Protection Volunteer
Age (years)	39.6	43.9
Sex (% female)	48.0%	47.3%
Education (years completed)	14.1	7.5
Years of experience in the position	15.6	9.4
Workload		
Number of families past month (Bh) or week (Rw)	20 in 1.8 groups	2.6
Months of experience delivering parenting pgm	4.1	2.0
Responsibilities outside parenting (h/week)	97% (11.0)	35.3% (6.1)
Feel overworked (%)	80.2%	8.0%
Feel respected and appreciated (%)	98.1%	100.0%
Training and Supervision		
Days trained	11.7	10.2
Refresher (%) (days since the last refresher)	12.3% (>30 days)	90.7% (<14 days)
Supervision (in-person) past 30 days	0.62%	85.3%
Convened with fellow workers for exchange and support (if yes, within the past 30 days)	31.1% (68.7%)	98.7% (92.3%)
Confidence in own delivery		
Immediate post-training (% 4 or 5 on a 5 pt scale)	60.4%	50.7%
Currently (% 4 or 5 on a 5 pt scale)	73.6%	88.0%
Self-rated quality of delivery (0 to 10)	7.3	7.9
Knowledge of Child Development Inventory (max 40)	22.5	18.5
10-item child development (max 20)	11.6	8.7
10-item parenting stimulation (max 20)	10.9	9.8

Notes. Confidence in own delivery if rated 4 (mostly confident) or 5 (very confident).

**Table 4:** Quality of delivery observations of Bhutan’s Prescription to Play.

Quality Item	% Observed			Mean (SD)
Specific Activity	1 = Not Observed	2 = Needs Improvement	3 = Very Good	
Ask about homework	3.4%	48.3%	48.3%	2.5 (0.6)
Introduce new play	0.0	18.3	81.7	2.8 (0.4)
Coach responsiveness	20.0	63.3	16.7	2.0 (0.6)
Demonstrate new play	8.3	36.7	55.0	2.5 (0.7)
Give parent opportunity	15.0	43.3	41.7	2.3 (0.7)
Coach new practice	21.7	40.0	38.3	2.2 (0.8)
Explain the benefits for the child	3.4	33.3	63.3	2.6 (0.6)
Use visual aids	25.0	51.7	23.3	2.0 (0.7)
Offer problem-solving	11.7	55.0	33.3	2.2 (0.6)
Review session messages	11.7	25.0	63.3	2.5 (0.7)
Mean of ten qualities	12.0%	41.5%	46.5%	2.36 (0.6)
**Delivery skills**	**1 = Poor**	**2 = Needs improvement**	**3 = Very good**	**Mean (SD)**
Well-prepared	0.0%	56.7%	43.3%	2.4 (0.5)
Covered the content	0.0	31.7	68.3	2.7 (0.5)
Lively and engaging	0.0	76.7	23.3	2.2 (0.4)
Acceptance and empathy	5.0	50.0	45.0	2.4 (0.6)
**Parent engagement**	**No**	**Few**	**Some**	**Most**
Parent did homework	13.3%	55.8%	36.5%	7.7%
Parent practices with the child during the session	10.0	51.9	40.7	7.4
Parent has own playthings	41.7	71.4	28.6	0.0

**Table 5:** Quality of delivery observations of Rwanda’s Sugira Muryango.

Quality Item	% Observed			Mean (SD)
Specific Activity	1 = Not Observed	2 = Needs Improvement	3 = Very Good	
Ask about homework	4.9%	29.0%	66.1%	2.6 (0.6)
Introduce new play	22.6	22.6	54.8	2.3 (0.8)
Coach responsiveness	22.6	29.0	48.4	2.3 (0.8)
Demonstrate new play	19.3	24.2	56.5	2.4 (0.8)
Give parent opportunity	24.1	19.4	56.5	2.3 (0.8)
Coach new practice	21.0	27.4	51.6	2.3 (0.8)
Explain the benefits for the child	14.5	29.0	56.5	2.4 (0.7)
Use visual aids	3.2	24.2	72.6	2.7 (0.5)
Offer problem-solving	11.3	25.8	62.9	2.5 (0.7)
Review session messages	8.1	27.4	64.5	2.6 (0.6)
Mean of ten qualities	15.2%	25.8%	59.0%	2.44 (0.7)
**Delivery skills**	**1 = Poor**	**2 = Needs improvement**	**3 = Very good**	**Mean (SD)**
Well-prepared	1.6%	35.5%	62.9%	2.6 (0.5)
Covered the content	1.6	32.3	66.1	2.6 (0.5)
Lively and engaging	1.6	27.4	71.0	2.7 (0.5)
Acceptance and empathy	1.6	22.6	75.8	2.7 (0.5)
**Parental engagement**	**No**	**Yes**		
Parent did homework	1.6%	98.4%		
Parent practices with child	8.1	91.9		
Parent has own playthings	8.1	91.9		

## Data Availability

Some anonymized data may be obtained from the authors.
